# Malaria-anemia comorbidity prevalence as a measure of malaria-related deaths in sub-Saharan Africa

**DOI:** 10.1038/s41598-019-47614-6

**Published:** 2019-08-05

**Authors:** Isidoros Papaioannou, Jürg Utzinger, Penelope Vounatsou

**Affiliations:** 1 Swiss Tropical and Public Health Institute, Basel, Switzerland; 2 University of Basel, Basel, Switzerland

**Keywords:** Malaria, Epidemiology

## Abstract

Different methods and data sources have been utilized to determine the relationship between malaria and mortality in endemic countries. Most of these efforts have focused on deaths directly attributed to malaria, while they overlooked causes of mortality that might be indirectly related to the disease, for instance anemia. We estimated the association of malaria parasitaemia, anemia, and malaria-anemia comorbidity with all-cause under-five mortality and evaluated the potential of malaria-anemia comorbidity prevalence to quantify malaria-related deaths in sub-Saharan Africa. We analysed data from Demographic and Health Surveys (DHS) and employed Bayesian geostatistical models. Mortality hazard obtained from malaria-anemia comorbidity prevalence was up to 3·5 times higher compared to the hazard related to *Plasmodium* parasitaemia only. Malaria parasite prevalence alone could not always capture a statistically important association with under-five mortality. Geographical variation of the malaria-anemia comorbidity effect was observed in most, but not all, countries. We concluded that the malaria burden in sub-Saharan Africa is considerably underestimated when anemia in not taken into account and that the malaria-anemia comorbidity prevalence provides a useful measure of the malaria-related deaths.

## Introduction

In 2016, malaria caused an estimated 216 million clinical episodes and 445,000 deaths^1^. Sub-Saharan Africa is the most afflicted region globally; indeed, 91% of all malaria deaths are concentrated in this part of the world. Children under the age of 5 years are at highest risk and might contribute up to 70% of all malaria-related deaths. Large-scale control efforts led to an 18% reduction in the incidence rate of malaria between 2000 and 2016 and six countries were certified by the World Health Organization (WHO) as malaria-free^1^. In 2016, the financial investment in malaria control and elimination was estimated at US$ 2·7 billion. Eliminating malaria by 2030 is part of the aspirations of the Sustainable Development Goals (SDGs)^2^. The 10 sub-Saharan African countries with the highest malaria burden experienced an increase in malaria incidence from 2015 to 2016, therefore the WHO recently launched the 10 + 1 initiative^3^ which aims to renew the focus on achieving the objectives set up by the Global Technical Strategy and Sustainable Development Goals.

Understanding the relation between malaria transmission and malaria-related mortality provides a useful insight in monitoring and evaluation of malaria control and elimination efforts. Furthermore, it helps in the planning of interventions since regions that are expected to have high malaria deaths could have interventions in place, such as availability of rapid diagnostic tests (RDTs) and Artemisinin-based combination therapy (ACT) in health facilities and information campaigns to prevent progression to severe malaria and therefore death. Several approaches and different data sources have been utilized to quantify malaria-related deaths. For example, the ‘Malaria Transmission Intensity and Mortality Burden across Africa’ (MTIMBA) project collected geo-referenced entomological data, which were linked with all-cause and malaria-specific mortality data derived from health and demographic surveillance systems (HDSS). A geostatistical analysis^4^ of the MTIMBA data from the Rufiji HDSS in Tanzania found no significant relation between neonate, infant, and 1- to 4-year-old child survival with malaria transmission, the latter measured by the entomological inoculation rate (EIR). In contrast, a 5-year collection of monthly data from the Kisumu HDSS in Kenya revealed a strong association between malaria-mortality and malaria transmission, the latter determined by the slide positivity rate (SPR)^5^. Malaria survey and mortality data from the Demographic and Health Surveys (DHS) linked to malaria incidence from the Malaria Atlas Project (MAP) suggested a 57% decrease of malaria-related mortality during 1990–2015^6^. However, analyses of DHS mortality data from Mali and Malawi with historical malaria survey data were not able to capture a statistically significant malaria-mortality relation^7,8^. A systematic analysis^9^ of malaria mortality data between 1980 and 2010 estimated 1·238 million malaria deaths globally in 2010.

Most of the studies estimating malaria-related deaths overlooked indirect causes of malaria mortality^10^. Indeed, one of the main outcomes of *Plasmodium* infection is anemia caused by the rupture of red blood cells as part of the complex life cycle^11^*. P. falciparum* leads to severe malarial anemia, which might be responsible for around one third of malaria deaths^12^. Righetti and colleagues^13^ reported a significant negative association between *Plasmodium* infections and haemoglobin concentrations. Another study reported stronger associations of anemia with malaria than iron deficiency or other nutritional, infectious, and genetic contributors^14^. Despite the proven association between anemia, malaria, and mortality, modelling studies quantifying the association between malaria-anemia comorbidity and under-five mortality are rather few.

We estimated the association of malaria parasitaemia, anemia, and malaria-anemia comorbidity with all-cause under-five mortality and evaluated the potential of malaria-anemia comorbidity prevalence to quantify malaria-related deaths in sub-Saharan Africa. We compiled available household-based data collected from DHS and Malaria Indicator Surveys (MIS) and employed a rigorous Bayesian geostatistical modeling framework. We estimated within-country variation of the association between comorbidity and under-5 mortality, using spatially varying coefficient models. Our results are presented at high spatial resolution, including model-based risk maps of malaria, anemia, and malaria-anemia comorbidity.

## Results

A total of 8,116 unique locations from 16 sub-Saharan Africa countries were included in this study. Of these, Angola, Benin, and Uganda have the highest number of locations. The earliest survey from which we extracted data is from Burkina Faso, conducted in 2010, while the latest is from Burundi, conducted in 2016–2017. Malaria prevalence among children under 5 years ranged from 2.2% in Rwanda to 76.1% in Burkina Faso. All countries of West Africa, except Senegal, had higher malaria prevalence compared to East African countries. The highest moderate/severe anemia prevalence was found in Burkina Faso (69.6%). Burkina Faso, Guinea, and Cameroon showed, in descending order, the highest under-five mortality rate. Despite relatively high malaria parasitaemia and anemia levels, Ghana revealed the second lowest under-five mortality rate among the 16 countries examined. Burkina Faso, Burundi, Mozambique, and Uganda were the four countries characterized by a higher malaria burden compared to the anemia burden. All countries except Burkina Faso (11.1%) had severe anemia levels below 10% with the lowest rate observed in Rwanda (0.7%). Overall, the mean malaria prevalence was 31.1%, mean moderate/severe anemia was 39.7%, and mean severe anemia was 3.7%.

**Figure 1:**
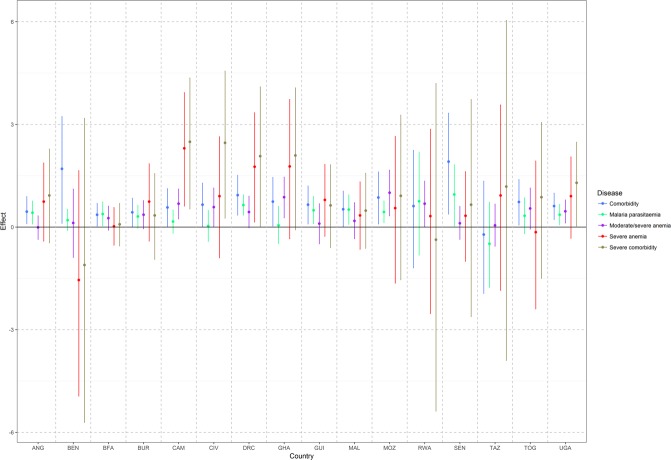
Bayesian estimates (posterior median, 95% BCI) of malaria parasitaemia, moderate/severe anemia (<100 g/l), severe anemia (<70 g/l), comorbidity, and severe comorbidity prevalence on under-five mortality in 16 sub-Sahara African countries. Models were adjusted for climate, maternal and household characteristics, malaria- anemia interventions, and individual child covariates. ANG, Angola; BEN, Benin; BFA, Burkina Faso; BUR, Burundi; CAM, Cameroon; CIV, Côte d’Ivoire; DRC, Democratic Republic of the Congo; GHA, Ghana; GUI, Guinea; MAL, Mali; MOZ, Mozambique; RWA, Rwanda; SEN, Senegal; TAZ, Tanzania; TOG, Togo; UGA, Uganda.

Our estimates (Fig. 1) highlight a statistically important effect of comorbidity on under-five mortality across all countries, except Rwanda and Tanzania. We identified a statistically important effect of malaria-anemia comorbidity on under-five mortality in 14 out of 16 countries (except Rwanda and Tanzania), while malaria prevalence alone was statistically important in half of the countries. In Benin, the hazard ratio (HR) of comorbidity was 3·5 times the hazard ratio of parasitaemia alone. The HR of comorbidity was approximately 50% higher in Cameroon, Mozambique, and Togo and approximately 30% higher in Uganda and the Democratic Republic of the Congo compared to the HR of parasitaemia alone. We observed the lowest difference in hazard ratios in Angola and Mali, with 7% and 3% higher malaria-anemia comorbidity hazard, respectively. Burkina Faso was the only country with an important comorbidity effect and no statistically important difference in the hazard ratios of comorbidity and parasitaemia.

Using the same set of confounders across all countries, we found a statistically important association of malaria-anemia comorbidity prevalence with under-five mortality in 9 out of 16 countries examined (Supplementary Appendix, Table 1.3). Similarly to the results above, the comorbidity prevalence was associated with mortality in countries which did not have an important parasitaemia-mortality relation. In countries with a significant parasitaemia-mortality association, the coefficient of the comorbidity was greater than that of parasitaemia (except Burkina Faso, as observed with country-specific covariates). Estimates for the comorbidity prevalence, adjusted for parasitaemia only prevalence, anemia only prevalence and country-specific confounders, were higher in 7 out of 11 countries compared to unadjusted models for parasitaemia and anemia only prevalences (Supplementary Appendix, Table 1.4). However, in the adjusted analyses the comorbidity was not statistically important in Angola, Guinea and Mali.

Severe disease expressed either as severe anemia or severe comorbidity has the highest burden on under-five mortality. As all countries have relatively low severe anemia prevalence, there is high uncertainty in our estimates, contributing to important associations only in 4 out of the 16 countries investigated (Fig. 1). However, when the severe comorbidity-mortality relation is captured, the HR can be more than five times higher to that of comorbidity, as observed in Cameroon and Côte d’Ivoire. Similarly, when we identify an important severe anemia-mortality association, the HR estimates of severe anemia are greater than the ones of comorbidity, albeit lower than that of severe comorbidity. Hence it can be inferred that severe disease has the greatest impact on under-five survival.

**Figure 2:**
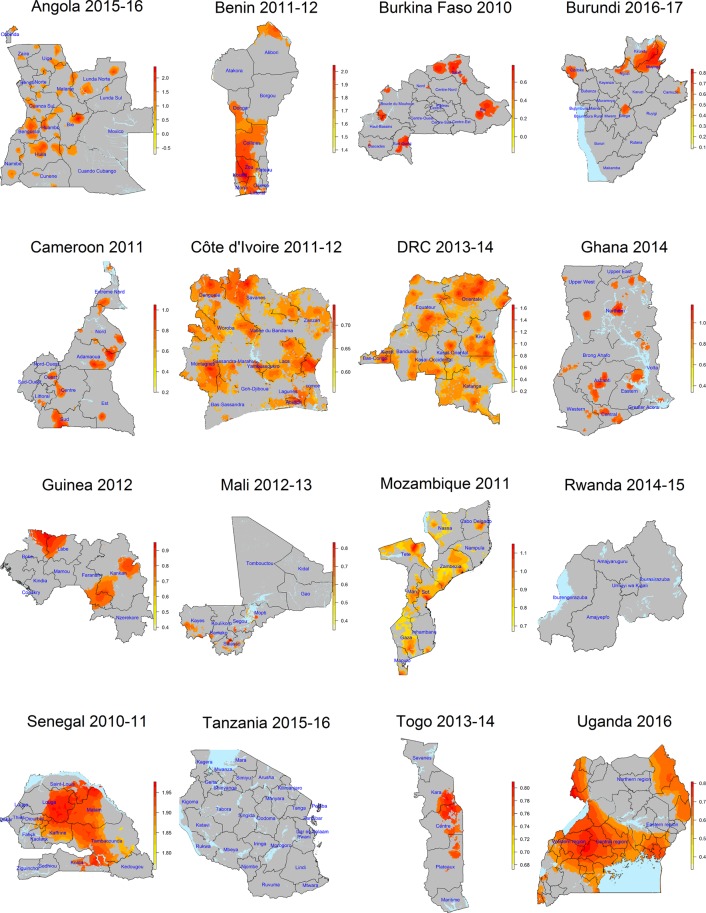
Posterior median of the malaria-anemia comorbidity effect on all-cause under-five mortality. Effects which are not statistically important (pixel-level posterior distribution includes zero) are indicated in grey colour.

Figure 2 demonstrates the geographical distribution of the comorbidity association with all-cause under-five mortality. All countries with a statistically important comorbidity effect at national level demonstrate subnational geographical variations. In some countries (i.e. Benin, Côte d’Ivoire, Democratic Republic of the Congo and Mozambique) there are large areas with a statistically important comorbidity effect. In others, (i.e. Angola, Burkina Faso, Burundi, Cameroon and Ghana) the effect is rather focal.

**Figure 3:**
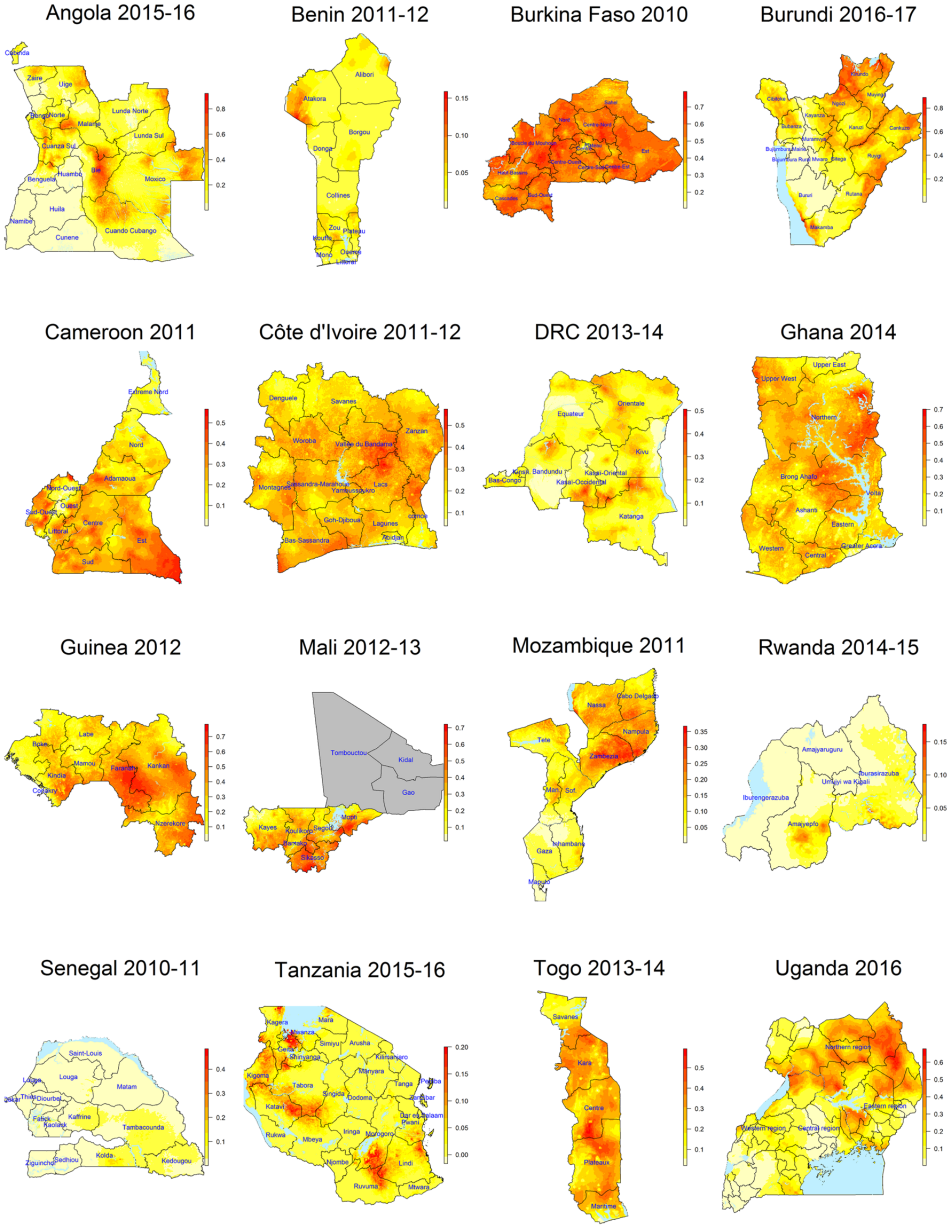
Posterior median of malaria parasitaemia and moderate or severe anemia comorbidity risk estimates at 2 × 2 km^2^ from Bayesian geostatistical models for 16 sub-Sahara African countries.

Model-based risk surfaces of the comorbidity effect on under-five mortality are presented in Fig. 3 and of malaria parasitaemia and anemia in the Supplementary Appendix. In some countries, we observed strong spatial patterns of moderate-to-severe anemia risk surfaces while in others there are only a few high risk hotspots. Noteworthy are areas in which high malaria parasitaemia risk coincides with densely populated surfaces, for example in northern Burundi, central-south Cameroon, central Guinea, and north-east Mozambique. Severe anemia-malaria maps refine all areas of high risk within a country and demonstrate regions of severe disease, i.e. areas between Lunda Sul and the Moxico border in Angola, Ngozi province in Burundi, Mopti province in Mali, the south-eastern part of Senegal, North Tanzania, and the south-western part of Côte d’Ivoire.

## Discussion

This is the first study assessing the association of malaria-anemia comorbidity with child mortality using routinely collected household survey data from DHS and MIS. We employed Bayesian geostatistical modeling and rigorous variable selection procedures to assess the geographical variation of the comorbidity effect on under-five mortality. We included in the analysis survey data with information on mortality, anemia and malaria at the same location to avoid potential bias from surveys misaligned in space and time.

Our findings demonstrate a strong association between under-five mortality and comorbidity burden, suggesting that higher prevalence of the comorbidity are related to higher mortality rates. Low malaria parasite prevalence, low under-five mortality, and moderate-to-high malaria intervention coverage were observed in countries with no statistically important comorbidity effects (i.e. Rwanda and Tanzania). In countries with a statistically important malaria-mortality relation, with the exception of Burkina Faso, the mortality hazard of comorbidity was higher than the mortality hazard of parasitaemia. A cross-sectional study in Rwanda found that children with moderate/severe anemia had a four times higher risk of malaria infection^15^. A prospective observational study in Gabon^16^ showed that moderate/severe malarial anemia was strongly associated with parasitaemia in children aged below four years. Other studies^17–21^ also suggested a strong relationship between malaria and anemia, irrespective of the malaria prevalence levels^22^. Our work demonstrates that moderate anemic children with malaria parasites have higher mortality hazards compared to those children with only parasitaemia.

When a common set of confounders was used across all models, we observed an important comorbidity-mortality association in fewer countries, albeit these countries did not have an important parasitaemia-mortality association. This modelling approach incorporates, in each model, unnecessary covariates and it does not take into account multicollinearity which can bias parameter estimation. The mortality-comorbidity association was not statistically important in Angola, Guinea and Mali when the model was adjusted for parasitaemia only and anemia only prevalences. In fact these countries have the highest anemia only prevalence (together with Senegal and Tanzania) which may explain the variation in mortality. We have adjusted for the malaria/anemia only to avoid high correlation with the comorbidity prevalence.

Among the factors investigated in our study, severe comorbidity has the highest impact on all-cause under-five mortality. Due to the low severe comorbidity prevalence, we obtained higher uncertainty in our estimates and thus we were only able to find an important association with under-five mortality in Cameroon, Côte d’Ivoire, Democratic Republic of the Congo, and Uganda. In these countries, the magnitude of association of severe comorbidity is far greater compared to that of other conditions and types of diseases examined. A study from the University Teaching Hospital in Ebonyi state in Nigeria showed that malaria accounted for approximately 77% of all children with severe anemia admission in the hospital^23^ and associated severe anemia with high under-five mortality rates. In western Kenya, an association of 85% between parasitaemia and severe anemia in hospital admissions is reported^24^, with severe anemia contributing to approximately half of malaria-related deaths. Severe comorbidity accounts for more than 30% in paediatric mortality^25^. Apart from profound results on children survival, severe malarial anemia could also be associated with long-term impairment in cognitive ability^26^.

Our findings revealed considerable geographical variation of the malaria-anemia comorbidity effect on all-cause under-five mortality in most countries. Several geographical areas with important comorbidity association coincide with known areas in which high mortality and low antimalarial treatment or ITN coverage exists^6^, for instance areas in Lunda Norte, Huila and Malanje provinces in Angola, Orientale, Katanga and Kivu provinces in Democratic Republic of the Congo, parts in north Cameroon and central Côte d’Ivoire, Faranan and Kankan provinces in Guinea and most of the areas shown in Mali. Additionally, some of the areas reside in provinces that experienced severe malaria burden at the year that the DHS-MIS was conducted, for instance the Orientale province in Democratic Republic of the Congo^27^.

In countries lacking a clear malaria-mortality relation, we found an important anemia-mortality association. Democratic Republic of the Congo, Mozambique, and Uganda are the only countries in this study showing that both malaria parasite and anemia prevalence are related to mortality. Greenwell and Neuman^28^ supported that anemia is an important indicator for monitoring malaria burden. Based on our findings, the malaria-anemia comorbidity indicator could be used to monitor severe disease.

Our work assessed the potential of anemia and malaria parasitaemia comorbidity prevalence to estimate malaria-related under-five mortality. Our findings confirm earlier results showing that malaria parasite prevalence alone fails to fully capture a statistically important association with mortality. Presence of malaria parasites is not directly linked to severe malaria that leads to death and thus statistical models may fail to capture the known association between malaria and mortality. In contrast, the comorbidity indicator can identify cases that experience severe disease, and hence, it is better related to mortality. It follows that the effect of comorbidity prevalence on all-cause under-five mortality can be a better indicator of malaria-related deaths than malaria prevalence alone, in survey data. Studies should further evaluate the use of comorbidity indicator in estimating malaria deaths for under-five children using malaria-specific mortality data.

Our work does not take into account changes in prevalence that might have been occurred over the last five years that mortality has been studied. This assumption may influence data stemming from the most recent surveys, when the scaling up of malaria interventions had already taken place. Yet, our analysis includes mostly surveys conducted before 2013, thus before the further scaling up of interventions. In our study the estimated effects are quantifying associations and by no means imply causal relations. Children with severe malaria anemia are less likely to participate in DHS or MIS, leading to underestimation of this effect.

## Methods

### Data sources

We analysed household representative survey data from 16 sub-Saharan African countries that had available malaria, anemia, and mortality data at survey locations. The surveys were conducted between 2010 and 2017. A summary of the data extracted from the DHS is provided in Table 1.

**Table 1:** Descriptive analysis of malaria survey data in 16 sub-Sahara African countries, collected from the DHS programme.

Country	Survey period	Number of locations	Malaria parasitaemia* (%)	Moderate/severe anemia* (<100 g/l)	Severe Anemia* (<70 g/l)	Moderate comorbidity*	Severe comorbidity*	ITN**	U5M***
Angola	10/2015–3/2016	625	13.1% (R)	34·0	2·2	10·1	1·5	31·0	68
Benin	12/2011–4/2012	747	28.4% (M)	32·1	3·0	10·9	1·0	79·8	70
Burkina Faso	1/2010–12/2010	542	76.1% (R)	69·6	11·1	53·2	8·5	56·9	129
Burundi	10/2016–3/2017	552	37.9% (R)	36·3	3·6	21·0	2·9	46·1	78
Cameroon	1/2011–8/2011	577	30.0% (R)	32·8	1·7	16·8	1·4	36·4	122
Côte d’Ivoire	12/2011–5/2012	341	41.5% (R)	49·7	3·3	27·7	2·4	67·3	108
DRC	8/2013–2/2014	492	22.6% (M)	27·1	2·3	11·9	1·4	70·0	104
Ghana	9/2014–12/2014	423	36.4% (R)	39·1	2·2	25·0	2·1	68·3	60
Guinea	6/2012–10/2012	300	43.9% (M)	52·4	7·6	27·0	4·9	47·0	123
Mali	11/2012–2/2013	413	51.6% (R)	60·8	9·3	33·4	6·7	84·4	95
Mozambique	5/2011–12/2011	610	38.3% (R)	26·6	1·4	21·7	2·7	51·4	97
Rwanda	11/2014–4/2015	492	2.2% (M)	15·7	0·7	1·5	0·2	80·6	50
Senegal	10/2010–4/2011	385	2.9% (M)	53·2	4·9	2·5	0·6	62·9	72
Tanzania	8/2015–2/2016	608	5.6% (M)	31·2	1·6	2·4	0·2	65·6	67
Togo	10/2013–4/2014	330	36.4% (M)	44·8	2·4	22·5	1·8	65·4	88
Uganda	6/2016–12/2016	679	30.3% (R)	29·2	2·3	17·1	1·8	78·4	64

*Representing prevalence; **Insecticide-treated net ownership; ***Under-five mortality (per 1,000); ****M for microscopy rest; R for RDT.

For each child, the mortality-related data consisted of the age and vital status (i.e. alive or dead) of the child. Furthermore, we considered in the analysis information on maternal, household and individual child characteristics. Maternal data included the educational attainment and literacy of the mother, age at pregnancy, delivery methods, other pregnancy terminations, and time intervals succeeding or preceding birth. Each child was classified into a socioeconomic category, using a household-based asset index which was already available in the data^29^. We also obtained information on the living standards of each child (e. g. improved sanitation facilities, improved drinking water sources, and open defecation practises). Individual child covariates included the sex of a child, birth order, breastfeeding practises of the mothers, and vaccination status.

Malaria and anemia data were extracted from readily available MIS. Some of the surveys used a combination of malaria microscopy and rapid diagnostic tests (RDTs) to diagnose malaria, while others used RDTs only. We classified the anemia level of a child based on guidelines put forward by the World Health Organization (WHO), as follows: (i) moderate or severe anemic if haemoglobin (Hb) levels were below 100 g/l and (ii) severe anemic if Hb levels were below 70 g/l. We also considered indicators of malaria intervention coverage at cluster level such as the proportion of households reporting indoor residual spraying (IRS), Artemisinin-based combination therapy coverage, the proportion of use or ownership of insecticide-treated nets (ITN), and the prevalence of iron supplementation in order to adjust for interventions. We defined comorbidity as malaria parasitaemia with moderate or severe anemia and severe comorbidity as parasitaemia with severe anemia. The DHS Program maintains strict standards for protecting the privacy of respondents and household members in all DHS surveys. Before each interview or biomarker test is conducted, an informed consent statement is read to the respondent, who may accept or decline to participate. Also, verbal informed consent for each parasitaemia test is provided by the child’s parent/guardian/caregiver on behalf of children less than 5 years before the test is conducted. Verbal consent is conducted by the interviewer reading a prescribed statement to the respondent and recording in the questionnaire whether or not the respondent consented or assent is provided. The interviewer signs his or her name attesting to the fact that he/she read the consent statement to the respondent. Written consent is not included (https://dhsprogram.com/publications/publication-dhsm7-dhs-questionnaires-and-manuals.cfm).

We extracted environmental and climatic data from remote sensing and other open access data sources. We downloaded from Moderate Resolution Imaging Spectroradiometer (MODIS) the normalized difference vegetation index (NDVI) and the land surface temperature (LST) at 1 × 1 km^2^ spatial resolution. We also obtained from MODIS the land cover type (LC) and distance from nearest water bodies (DWATER) at 0.5 × 0.5 km^2^ spatial resolution. LC was recoded to the following categories: forests, grasslands, and croplands. Rainfall data were obtained from the U.S. Geological Survey-Earth Resources Observation Systems (USGSS) Data Portal, while altitude data were extracted from the Shuttle Radar Topography Mission (SRTM) at 0.5 × 0.5 km^2^ spatial resolution. Locations were classified to rural or urban residence according to the Global Rural and Urban Mapping Project.

### Statistical analysis

We fitted separate Bayesian geostatistical Weibull survival models, for each one of the morbidity indicators considered in our study, i.e. malaria parasitaemia, moderate/severe anemia, severe anemia, moderate comorbidity and severe comorbidity, in order to assess their effect on all-cause under-five mortality. The outcome of the survival model was the age of death or current age of a child (in months), together with the corresponding censoring status, i.e. alive children were considered as censored observations. The indicators measured cluster-level prevalence and thus each child was associated with the prevalence at the corresponding cluster. Therefore, the prevalence of malaria parasitaemia, anemia, and malaria-anemia comorbidity at a given location was treated as an exposure that children receive at that location. For each model, the association between the mortality outcome and the corresponding indicator was adjusted for confounders, which were selected based on rigorous variable selection procedures, carried out separately for each country. In particular, we fitted separate Weibull survival geostatistical models with the candidate covariates and included only the statistically important ones (i.e. Bayesian credible interval did not include zero). We used throughout the paper terminology consistent with the Bayesian inference and call statistically important effects regression coefficients that are known as statistically significant in frequentist inference. The variance inflation factor was used to exclude the highly correlated predictors^30^. The final models included different sets of covariates for each country in order to estimate the malaria parasitaemia, anemia, and comorbidity effect from the most parsimonious model. We included spatially random effects at cluster level modelled by a Gaussian process with Matérn covariance matrix^31^.

An additional analysis was carried out to assess the sensitivity of the results to the selected confounders. Specifically, we re-fitted the parasitaemia and comorbidity prevalence models using a common set of confounders across all countries based on the biggest set derived by combining all country-specific confounders. Furthermore, we re-fitted the models with the comorbidity prevalence adjusting for parasitaemia prevalence only (prevalence of children with positive parasite test which are not moderate or severe anemic) and moderate-to-severe anemia prevalence only (prevalence of moderate or severe anemic children which are not positive for a malaria parasite test).

We identified the geographical distribution in the effects of moderate comorbidity on all-cause under-five mortality, by fitting a spatially varying coefficient model for each country, assuming a spatially continuous Gaussian process on the comorbidity effect. Estimates were summarised by the posterior median, 2·5 and 97·5 quantiles. The models were adjusted for malaria or anemia interventions, climatic, maternal, individual child, and household characteristics.

We used geostatistical models to estimate disease risk surfaces and created national maps based on pixel-based estimates. Environmental predictors and spatial random effects were modelled on the logit scale. Prediction was carried out into a grid formed by 2 × 2 km^2^ resolution pixels.

The analysis was conducted in R software (version 3.3.2). We used the integrated nested Laplace approximations (INLA) approach^32^ to perform fast approximate Bayesian inference. Model details are provided in Supplementary Appendix.

### Ethical approval and consent to participate

In this study we analysed secondary data made available by the Demographic Health Survey (DHS) MEASURE. According to survey protocols and related documents of the surveys, ethical approval was obtained from the Institutional Review Board of International Consulting Firm (ICF) of Calverton, Maryland, USA, and also from the national ethical committees in the countries that the surveys were contacted. Details of ethical clearance are published in the DHS reports available at https://dhsprogram.com/publications/index.cfm.

## Supplementary information

Supplementary Appendix

## Data Availability

The study data are available upon request from the Demographic and Health Surveys program (https://dhsprogram.com/).

## References

[1] WHO. *World Malaria Report 2017*. (World Health Organization, Geneva, 2017).

[2] UN Department of Economic and Social Affairs. *Sustainable Development Goals*., https://sustainabledevelopment.un.org/topics/sustainabledevelopmentgoals (2018).

[3] WHO. *WHO Malaria Policy Advisory Committee (*MPAC*) meeting. Meeting Report*. (World Health Organization, Geneva, 2018).

[4] Rumisha SF, Smith TA, Masanja H, Abdulla S, Vounatsou P (2014). Relationship between child survival and malaria transmission: an analysis of the malaria transmission intensity and mortality burden across Africa (MTIMBA) project data in Rufiji demographic surveillance system, Tanzania. Malar J..

[5] Khagayi S, Amek N, Bigogo G, Odhiambo F, Vounatsou P (2017). Bayesian spatio-temporal modeling of mortality in relation to malaria incidence in Western Kenya. PLoS ONE.

[6] Gething PW (2016). Mapping Plasmodium falciparum Mortality in Africa between 1990 and 2015. N Engl J Med..

[7] Gemperli A, Vounatsou P, Sogoba N, Smith T (2006). Malaria mapping using transmission models: application to survey data from Mali. Am J Epidemiol..

[8] Kazembe LN, Appleton CC, Kleinschmidt I (2007). Spatial analysis of the relationship between early childhood mortality and malaria endemicity in Malawi. Geospat Health..

[9] Murray CJ (2012). Global malaria mortality between 1980 and 2010: a systematic analysis. Lancet..

[10] WHO. *WHO Malaria Policy* Advisory *Committee (MPAC) meeting. Meeting Report*. (World Health Organization, Geneva, 2017).

[11] Kai OK, Roberts DJ (2008). The pathophysiology of malarial anaemia: where have all the red cells gone?. BMC Med..

[12] Haldar, K. & Mohandas, N. Malaria, erythrocytic infection, and anemia. *Hematology Am Soc Hematol Educ Program*. 87–93 (2009). 10.1182/asheducation-2009.1.87 PMC2933134 20008186

[13] Righetti AA (2013). Dynamics of Anemia in Relation to Parasitic Infections, Micronutrient Status, and Increasing Age in South-Central Côte d’Ivoire. J Infect Dis..

[14] Foote EM (2013). Determinants of Anemia among Preschool Children in Rural, Western Kenya. Am J Trop Med Hyg..

[15] Kateera F (2015). Malaria, anaemia and under-nutrition: three frequently co-existing conditions among preschool children in rural Rwanda. Malar J..

[16] Bouyou-Akotet MK (2009). Impact of Plasmodium falciparum infection on the frequency of moderate to severe anaemia in children below 10 years of age in Gabon. Malar J..

[17] Osterbauer B (2012). Factors associated with malaria parasitaemia, malnutrition, and anaemia among HIV-exposed and unexposed Ugandan infants: a cross-sectional survey. Malar J..

[18] Magalhães RJS (2013). Role of malnutrition and parasite infections in the spatial variation in children’s anaemia risk in northern Angola. Geospat Health..

[19] Ceesay SJ (2015). Malaria Prevalence among Young Infants in Different Transmission Settings, Africa. Emerg Infect Dis..

[20] Moraleda C (2017). Anaemia in hospitalised preschool children from a rural area in Mozambique: a case control study in search for aetiological agents. BMC Pediatr..

[21] Menon MP, Yoon SS (2015). Prevalence and Factors Associated with Anemia among Children under 5 Years of Age—Uganda, 2009. Am J Trop Med Hyg..

[22] McCuskee S, Brickley EB, Wood A, Mossialos E (2014). Malaria and Macronutrient Deficiency as Correlates of Anemia in Young Children: A Systematic Review of Observational Studies. *Ann*. Glob Health.

[23] Muoneke VU, Ibekwe RC, Nebe-Agumadu HU, Ibe BC (2012). Factors associated with mortality in under-five children with severe anemia in Ebonyi, Nigeria. Indian Pediatr..

[24] Obonyo CO, Vulule J, Akhwale WS, Grobbee DE (2007). In-hospital morbidity and mortality due to severe malarial anemia in western Kenya. Am J Trop Med Hyg..

[25] Perkins DJ (2011). Severe malarial anemia: innate immunity and pathogenesis. Int J Biol Sci..

[26] Bangirana P (2014). Severe malarial anemia is associated with long-term neurocognitive impairment. Clin Infect Dis..

[27] DOCTORS WITHOUT BORDERS. *DRC: Urgent Action Needed to Prevent Malaria Deaths in Orientale Province*, https://www.doctorswithoutborders.org/news-stories/press-release/drc-urgent-action-needed-prevent-malaria-deaths-orientale-province (2018).

[28] Greenwell, F. & Neuman, M. *Children’s anemia levels in West Africa: a good proxy for malaria morbidity?* http://paa2006.princeton.edu/papers/61268 (2018).

[29] Rutstein, S.O. & Johnson, K. *The DHS Wealth Index. DHS Comparative Reports No. 6*. (ORC Macro, Calverton, Maryland, 2004).

[30] O’Brien RM (2007). A Caution Regarding Rules of Thumb for Variance Inflation Factors. Qual Quant..

[31] Banerjee, S., Carlin, B. P. & Gelfand, A. E. *Hierarchical Modeling and Analysis for Spatial Data, Second Edition*. (Chapman and Hall/CRC, Boca Raton, Florida, 2014).

[32] Rue H, Martino S, Chopin N (2009). Approximate Bayesian inference for latent Gaussian models by using integrated nested Laplace approximations. J R Stat Soc Series B Stat Methodol..

