# Supplementary material for: Malaria-anemia comorbidity prevalence as a measure of malaria-related deaths in sub-Saharan Africa
Source: Sci Rep. 2019 Aug 5;9:11323. doi: 10.1038/s41598-019-47614-6 (PMC6683112; doi:10.1038/s41598-019-47614-6)

Supplementary Appendix

Supplement to: Papaioannou, I., Utzinger, J. & Vounatsou, P.

Malaria-anemia comorbidity prevalence as a measure of malaria-related deaths in sub-Saharan Africa.

Contents

1 Supplementary material

1.1 Model specification

1.2 Bayesian estimates (posterior median, 95% BCI) of malaria, anemia, and malaria-anemia comorbidity prevalence on under-five mortality. Models were adjusted for country-specific confounders related to climate, mother, individual child and household characteristics as well as malaria- anemia interventions.

1.3 Bayesian estimates (posterior median, 95% BCI) of malaria parasitaemia and malaria-anemia comorbidity prevalence on under-five mortality. Models were adjusted for a common set of confounders based on the biggest set derived by combining all country-specific confounders.

1.4 Bayesian estimates (posterior median, 95% BCI) of malaria-anemia comorbidity on under-five mortality. Models were adjusted for parasitaemia only prevalence (children without moderate/severe anemia), anaemia only prevalence (children without malaria parasitaemia) and country-specific confounders.

1.5 Bayesian geostatistical model-based malaria parasitaemia risk estimates in 16 sub-Sahara African countries at 2x2 km^2.

1.6 Bayesian geostatistical model-based moderate/severe anemia risk estimates in 16 sub-Sahara African countries at 2x2 km^2.

1.7 Bayesian geostatistical model-based severe anemia risk estimates in 16 sub-Sahara African countries at 2x2 km^2.

1.8 Bayesian geostatistical model-based severe comorbidity risk estimates in 16 sub-Sahara African countries at 2x2 km^2.

1 Supplementary material

1.1 Model specification

1.1.1 Bayesian geostatistical Weibull survival model

We developed a geostatistical Weibull survival model to assess the association of malaria, anemia, and malaria-anemia comorbidity with under-five mortality in 16 sub-Saharan Africa countries. Let $s = \{s_1, s_2, \ldots, s_n\},\ s_i \subset R^2$ be the set of surveyed locations with observed malaria, anemia, and mortality data. We defined T_{ji} the age of death for the j-th child at location s_i and X_{ji} the row-vector of the associated covariates. We assumed a Weibull lifetime distribution for time T_{ji} with shape parameter α and scale parameter λ and probability density function $f(t_{ji}) = \alpha\, t_{ji}{}^{\alpha-1}\, \lambda \exp(-\lambda\, t_{ji}{}^{\alpha}), \alpha > 0, \lambda > 0$. Under this specification, the

corresponding hazard $h(t)$ and survival $S(t)$ have the following form: $h(t_{ji}) = a\, t_{ji}{}^{a-1}\,\lambda$ and $S(t_{ji}) = \exp(-\lambda t_{ji}{}^{\alpha})$. We introduced predictors on the scale parameter using a log link function, that is: $\lambda_{ji} = \exp(\eta_{ji})$, where η_{ji} is the linear predictor defined by the equation,

$$\eta_{ji} = \beta_0 + \sum_{\kappa=1}^{K} \beta_\kappa x_{ji\kappa} + \xi_i,$$

where $\beta = (\beta_0, \beta_1, \dots, \beta_K)^T$ is the vector of K regression coefficients, $x_{ji\kappa}$ represents the value of the κ-th predictor and $\xi(s) = (\xi_1, \xi_2, \dots, \xi_n)^\tau$ are multivariate Normal distributed random variables that take into account for spatial correlation in the response among the s locations. The multivariate Normal distribution followed a zero-mean specification with covariance between locations s_1 and s_2 described by the Matérn function, i.e. $\xi(s) \sim N(0, \Sigma_1)$ and $\Sigma_1(s_1, s_2) = \frac{\sigma_1^2 (\kappa_1\, d(s_1,s_2))^\nu K_\nu(\kappa_1\, d(s_1,s_2))}{\Gamma(\nu)\, 2^{\nu-1}}$, with σ_1^2 being the spatial process variance, $d(s_1, s_2)$ the distance between locations s_1 and s_2 and κ_1 the scaling parameter. K_ν is the modified Bessel function of second kind and order ν. Spatial range (r_1) describes the distance at which the spatial correlation becomes negligible and under the Matérn specification is defined as $r_1 = \frac{\sqrt{8}}{\kappa_1}$. We selected normally distributed priors $N(0,10)$ for regression coefficients and the default priors of INLA for the spatial hyperparameters and the shape of the Weibull distribution.

1.1.2 Spatially varying coefficient modelled via a spatially continuous Gaussian process

The above model was extended to include a spatially varying comorbidity effect. In particular, the linear predictor was written as follows:

$$\eta_{ji} = \beta_0 + b_i x_{ji1} + \sum_{\kappa=2}^{K} \beta_\kappa x_{ji\kappa} + \xi_i,$$

where b_i represents a spatially continuous Gaussian process, $\boldsymbol{b} \sim N(\beta_1, \Sigma_2)$ and $\boldsymbol{b} = (b_1, \dots, b_n)^\tau$. The mean β_1 represents the global comorbidity effect (i.e. at national scale). Σ_2 is a Matérn covariance function with spatial process variance σ_2^2 and scaling parameter κ_2 (section 1.1.1). The geographical distribution of the comorbidity effect was estimated by predicting the Gaussian process over a gridded surface of 2x2 km^2 spatial resolution.

1.1.3 Bayesian geostatistical binomial model

We developed Bayesian geostatistical binomial models to estimate disease risk surfaces, i.e. malaria parasitaemia, moderate/severe anemia, severe anemia, comorbidity, and severe comorbidity. We defined Y_i as the number of diseased under-five children at location s_i, $s = (s_1, s_2, \dots, s_n)^\tau$, $s_i \subset R^2$ and N_i the total number of children observed at that location. We assumed that for each location s_i the number of positive to the disease children Y_i follows a binomial distribution, i.e., $Y_i \mid N_i, \pi_i \sim Bin(N_i, \pi_i)$, with π_i being the disease risk at that location. The disease risk π_i was reparameterised in terms of predictor variables and regression parameters by using the logit link function as follows:

$$logit(\pi_i) = \beta^\tau X_i + \varphi_i,$$

where X_i is a set of environmental predictors observed at location s_i and $\beta = (\beta_0, \beta_1, \dots, \beta_k)^T$ the corresponding regression coefficients. The latent spatial process $\varphi(s) = (\varphi_1, \varphi_2, \dots, \varphi_n)^\tau$ follows the same specification as defined above.

1.1.4. Environmental and climatic data

Data	Source	Spatial resolution
Annual average Normalised Difference Vegetation Index (NDVI)	MODIS	1x1 km^2
Annual average Day and Night Land Surface Temperature (LST)	MODIS	1x1 km^2
Land Cover Type (LC)	MODIS	0·5x0·5 km^2
Distance from water bodies (DWATER)	MODIS	0·5x0·5 km^2
Annual average Rainfall	USGSS	8x8 km^2
Altitude (Digital Elevation model)	SRTM	0·5x0·5 km^2
Urban rural extent	GRUMP	1x1 km^2

MODIS: Moderate Resolution Imaging Spectroradiometer; USGSS: U.S. Geological Survey-Earth Resources Observation Systems; SRTM: Shuttle Radar Topographic Mission; GRUMP: Global Rural and Urban Mapping project

1.2 Bayesian estimates (posterior median, 95% BCI) of malaria, anemia, and malaria-anemia comorbidity prevalence on under-five mortality. Models were adjusted for country-specific confounders related to climate, mother, individual child and household characteristics as well as malaria- anemia interventions.

Condition<br><br>Country	Malaria parasitaemia	Moderate/ severe anemia	Severe anemia	Malaria-moderate/ severe anemia	Malaria/ severe anemia
Angola	**0·42<br>(0·09,0·76)**	-0·01<br>(-0·36,0·33)	0·74<br>(-0·41,1·87)	**0·45<br>(0·1,0·89)**	0·92<br>(-0·46,2·28)
Benin	0·20<br>(-0·10,0·52)	0·12<br>(-0·89,1·11)	-1·55<br>(-4·94,1·65)	**1·70<br>(0·11,3·23)**	-1·11<br>(-5·71,3·18)
Burkina Faso	**0·38<br>(0·04,0·73)**	0·26<br>(-0·09,0·61)	0·02<br>(-0·53,0·57)	**0·36<br>(0·03,0·69)**	0·08<br>(-0·55,0·69)
Burundi	0·31<br>(-0·03,0·63)	0·36<br>(-0·05,0·77)	0·74<br>(-0·41,1·85)	**0·43<br>(0·01,0·84)**	0·34<br>(-0·94,1·56)
Cameroon	0·16<br>(-0·18,0·50)	**0·68<br>(0·24,1·11)**	**2·30<br>(0·61,3·93)**	**0·57<br>(0·02,1·12)**	**2·49<br>(0·53,4·36)**
Côte d'Ivoire	0·03<br>(-0·42,0·49)	**0·58<br>(0·02,1·14)**	0·90<br>(-0·90,2·64)	**0·65<br>(0·02,1·28)**	**2·46<br>(0·26,4·56)**
DRC	**0·64<br>(0·35,0·94)**	0·44<br>(-0·02,0·90)	**1·76<br>(0·15,3·34)**	**0·93<br>(0·35,1·51)**	**2·07<br>(0,4·10)**
Ghana	0·05<br>(-0·48,0·60)	**0·87<br>(0·27,1·46)**	1·77<br>(-0·34,3·73)	**0·74<br>(0·03,1·45)**	2·09<br>(-0·08,4·07)
Guinea	**0·49<br>(0·10,0·89)**	0·10<br>(-0·49,0·68)	0·79<br>(-0·27,1·83)	**0·65<br>(0·09,1·20)**	0·63<br>(-0·60,1·82)
Mali	**0·51<br>(0·08,0·94)**	0·18<br>(-0·34,0·71)	0·34<br>(-0·65,1·32)	**0·52<br>(0·01,1·05)**	0·48<br>(-0·62,1·57)
Mozambique	**0·44<br>(0·13,0·76)**	**1·00<br>(0·33,1·66)**	0·55<br>(-1·64,2·65)	**0·86<br>(0·09,1·61)**	0·91<br>(-1·54,3·27)
Rwanda	0·75<br>(-0·82,2·19)	**0·68<br>(0·01,1·34)**	0·32<br>(-2·53,2·86)	0·61<br>(-1·19,2·24)	-0·37<br>(-5·38,4·20)
Senegal	**0·95<br>(0·03,1·82)**	0·11<br>(-0·36,0·60)	0·33<br>(-1·00,1·62)	**1·91<br>(0·38,3·33)**	0·65<br>(-2·62,3·73)
Tanzania	-0·49<br>(-1·76,0·72)	0·05<br>(-0·56,0·67)	0·92<br>(-1·85,3·57)	-0·22<br>(-1·94,1·34)	1·18<br>(-3·90,6·04)
Togo	0·33<br>(-0·19,0·85)	0·54<br>(-0·06,1·14)	-0·15<br>(-2·39,1·93)	**0·73<br>(0·06,1·39)**	0·87<br>(-1·50,3·06)
Uganda	**0·36<br>(0·07,0·66)**	**0·46<br>(0·12,0·79)**	0·90<br>(-0·33,2·05)	**0·61<br>(0·22,0·99)**	**1·29<br>(0·03,2·48)**

1.3 Bayesian estimates (posterior median, 95% BCI) of malaria parasitaemia and malaria-anemia comorbidity prevalence on under-five mortality. Models were adjusted for a common set of confounders based on the biggest set derived by combining all country-specific confounders.

Condition<br><br>Country	Malaria parasitaemia	Malaria-moderate/ severe anemia
Angola	0.23<br>(-0.11,0.57)	0·21<br>(-0·24,0·66)
Benin	0.20<br>(-0.10,0.50)	**1.70**<br>**(0.15,3.19)**
Burkina Faso	**0.46**<br>**(0.12,0.81)**	**0.42**<br>**(0.09,0.75)**
Burundi	**0.34**<br>**(0.02,0.66)**	**0.43**<br>**(0.01,0.83)**
Cameroon	0.17<br>(-0.16,0.51)	0.48<br>(-0.07,1.02)
Côte d'Ivoire	0.24<br>(-0.18,0.68)	**0.87**<br>**(0.26,1.47)**
DRC	**0.70**<br>**(0.41,0.99)**	**1.04**<br>**(0.47,1.60)**
Ghana	0.10<br>(-0.41,0.61)	0.64<br>(-0.02,1.29)
Guinea	**0.62**<br>**(0.24,1.00)**	**0.91**<br>**(0.39,1.43)**
Mali	0.32<br>(-0.08,0.71)	0.33<br>(-0.16,0.82)
Mozambique	0.42<br>(-0.20,1.04)	0.63<br>(-0.16,1.39)
Rwanda	0.93<br>(-0.63,2.32)	0.77<br>(-1.02,2.36)
Senegal	**1.14**<br>**(0.25,1.98)**	**1.85**<br>**(0.40,3.20)**
Tanzania	-0.65<br>(-1.91,0.53)	-0.54<br>(-2.24,1.01)
Togo	0.45<br>(-0.06,0.95)	**0.78**<br>**(0.13,1.41)**
Uganda	0.29<br>(-0.01,0.57)	**0.55**<br>**(0.16,0.93)**

1.4 Bayesian estimates (posterior median, 95% BCI) of malaria-anemia comorbidity on under-five mortality. Models were adjusted for parasitaemia only prevalence (children without moderate/severe anemia), anaemia only prevalence (children without malaria parasitaemia) and country-specific confounders.

Condition<br><br>Country	Malaria-moderate/ severe anemia
Angola	0.31<br>(-0.16,0.78)
Benin	**1.78**<br>**(0.19,3.31)**
Burkina Faso	**0.64**<br>**(0.12,1.18)**
Burundi	**0.44**<br>**(0,0.87)**
Cameroon	**0.70**<br>**(0.12,1.26)**
Côte d'Ivoire	**0.65**<br>**(0.01,1.30)**
DRC	**0.75**<br>**(0.14,1.35)**
Ghana	**0.85**<br>**(0.11,1.57)**
Guinea	0.43<br>(-0.22,1.06)
Mali	0.42<br>(-0.17,1.00)
Mozambique	**1.03**<br>**(0.24,1.80)**
Rwanda	0.50<br>(-1.30,2.12)
Senegal	**1.89**<br>**(0.35,3.32)**
Tanzania	-0.11<br>(-1.83,1.46)
Togo	**0.72**<br>**(0.01,1.42)**
Uganda	**0.64**<br>**(0.24,1.03)**

1.5 Bayesian geostatistical model based malaria parasitaemia risk estimates in 16 sub-Sahara African countries at 2x2 km^2.

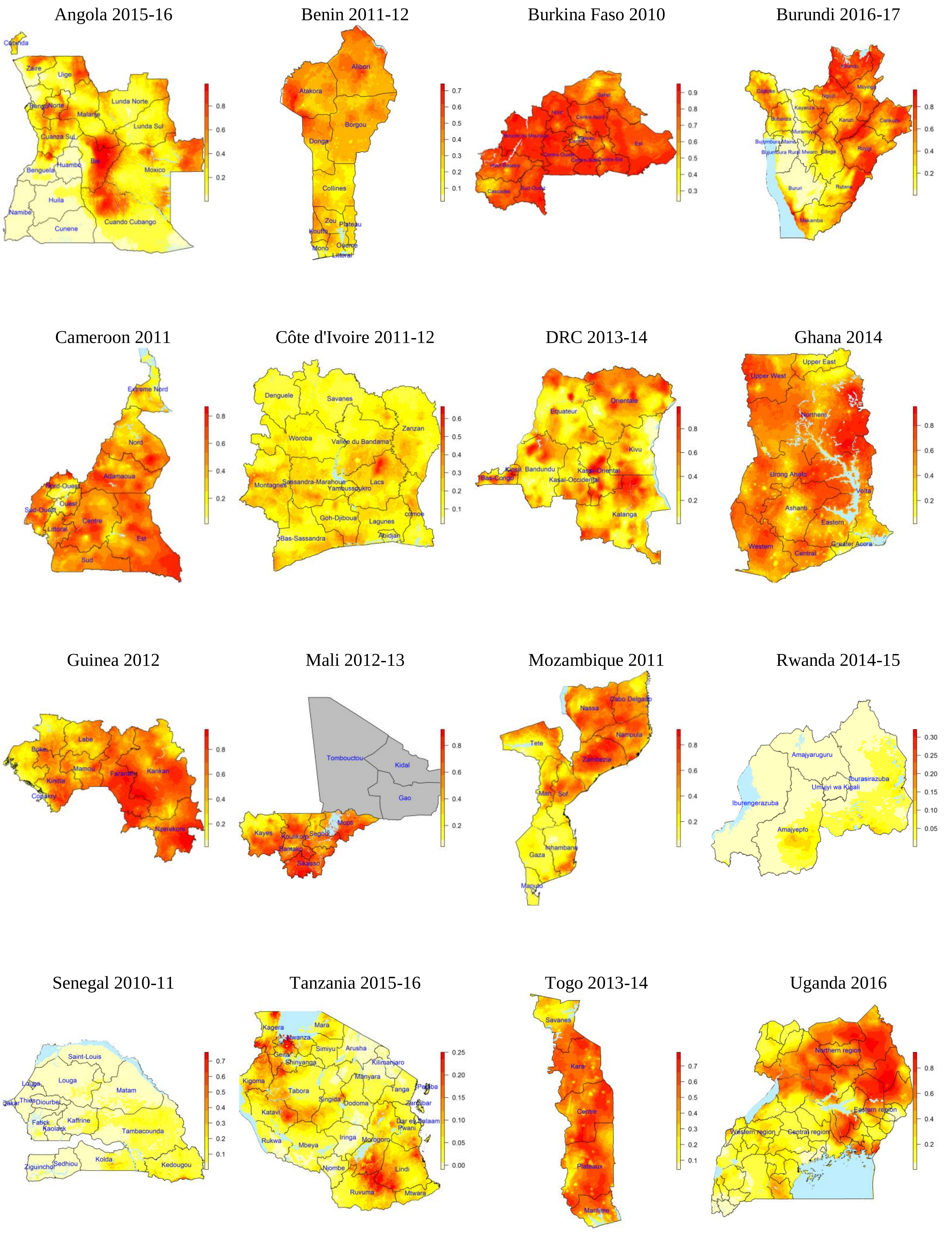

1.6 Bayesian geostatistical model based moderate/severe anemia risk estimates in 16 sub-Sahara African countries at 2x2 km^2.

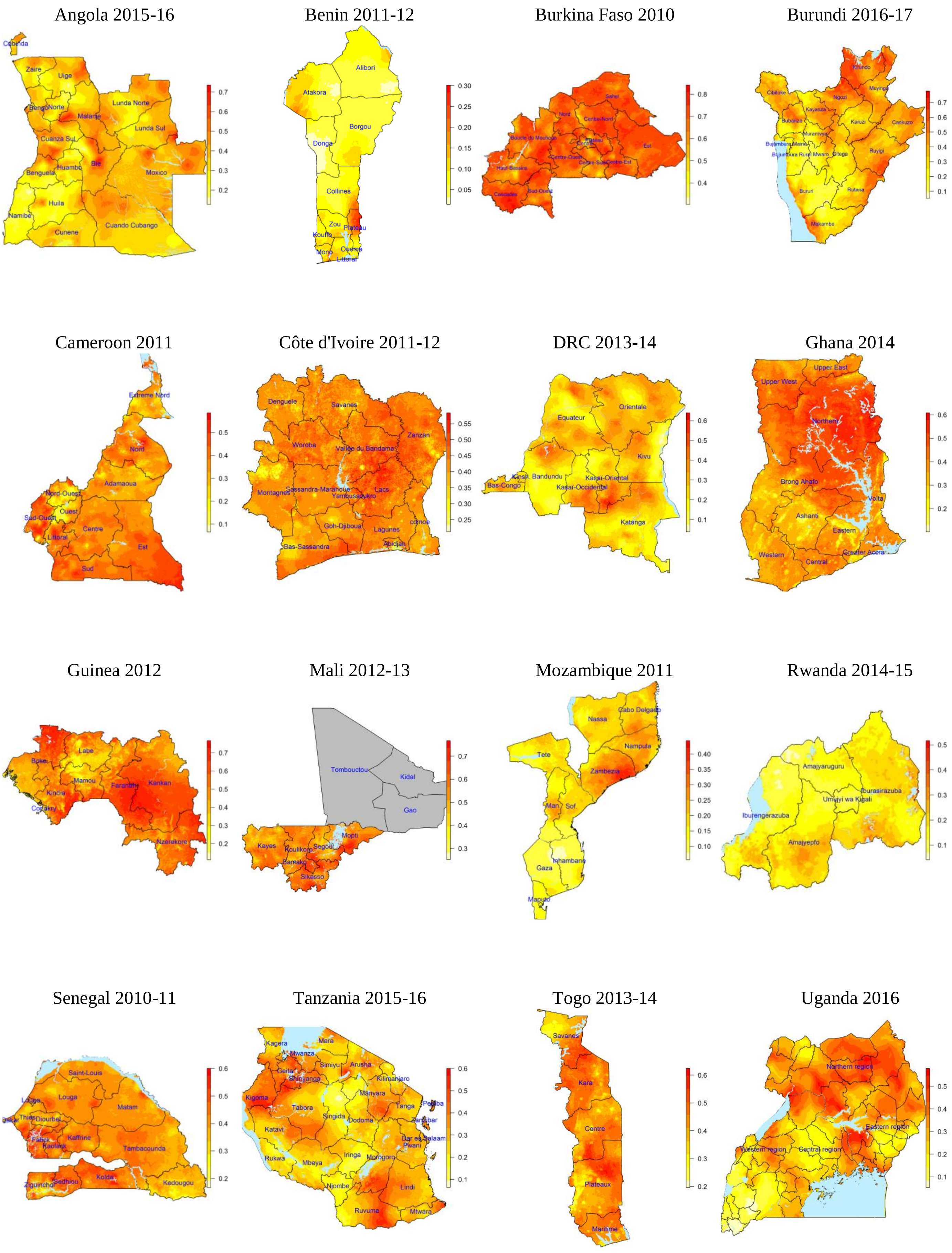

1.7 Bayesian geostatistical model based severe anemia risk estimates in 16 sub-Sahara African countries at 2x2 km^2.

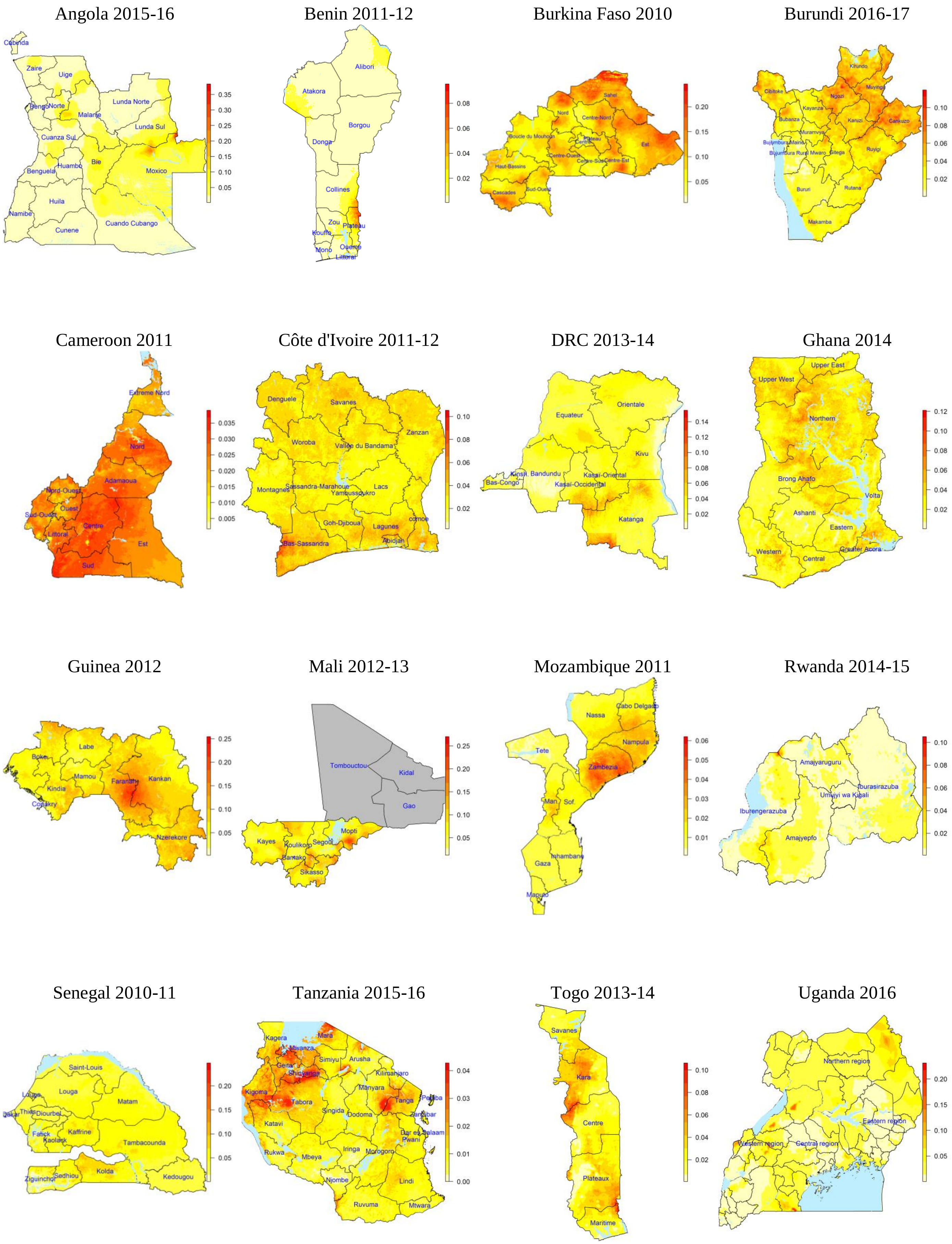

1.8 Bayesian geostatistical model based severe comorbidity risk estimates in 16 sub-Sahara African countries at 2x2 km^2.

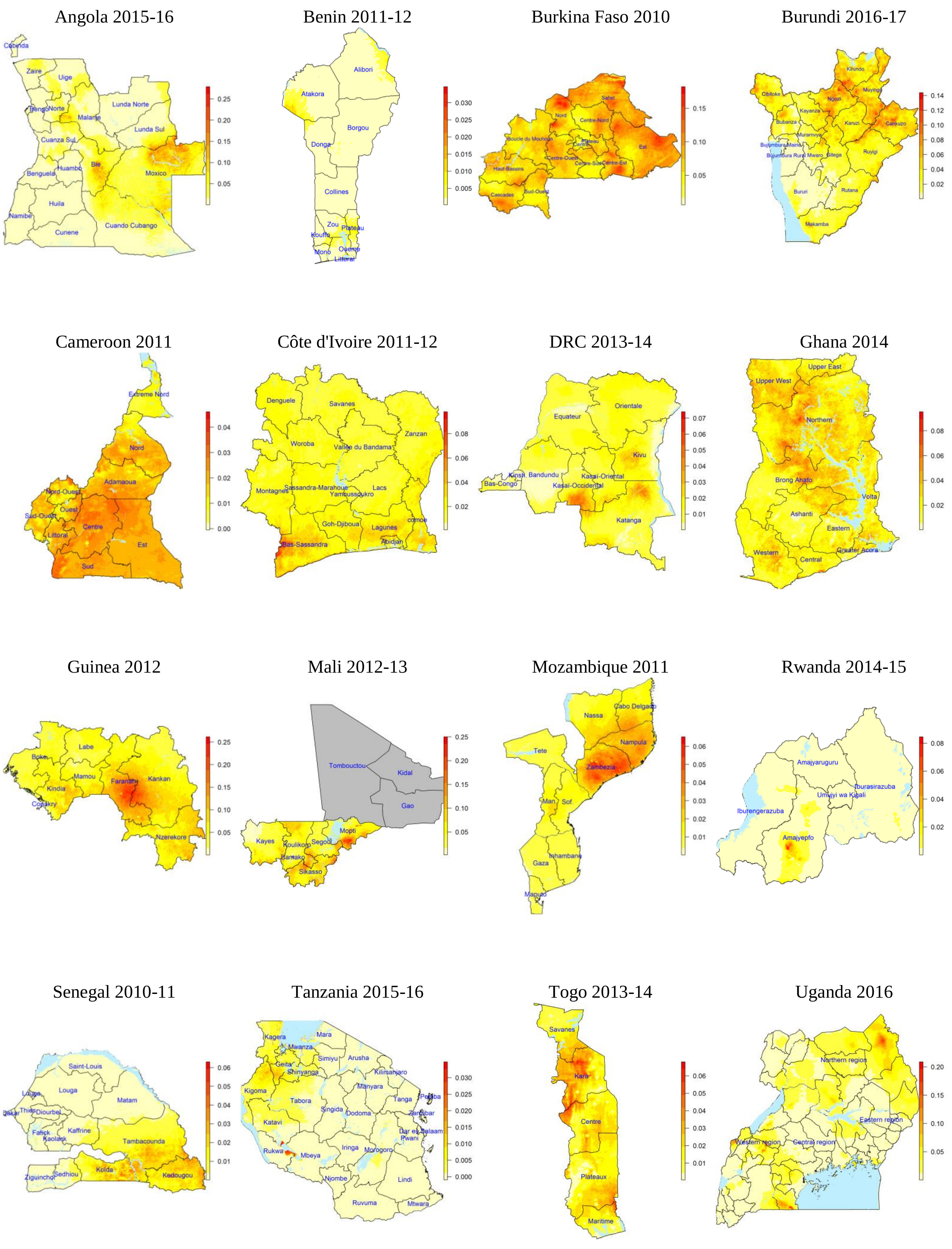

Supplement: Supplementary file 1 — Supplementary Appendix [file 41598_2019_47614_MOESM1_ESM.pdf]
